# Supplementary material for: Estimating epidemiological parameters of a stochastic differential model of HIV dynamics using hierarchical Bayesian statistics
Source: PLoS One. 2018 Jul 25;13(7):e0200126. doi: 10.1371/journal.pone.0200126 (PMC6059410; doi:10.1371/journal.pone.0200126)
Supplement: S2 File — Details of the model fit probabilities for each year and simulation. (PDF) [file pone.0200126.s002.pdf]

Undiagnosed

Year	Base Model	Exhaustion of Susceptibles	ART Usage	Lack of Access to Care	ES & ART	ES & LAC	LAC & ART	ES & LAC & ART
2005	0.9962	0.9962	0.9962	0.9962	0.9962	0.9962	0.9962	0.9962
2006	0.9116	0.9116	0.9116	0.9116	0.9116	0.9116	0.9116	0.9116
2007	0.8891	0	0.9018	0.9084	0	0.2507	0.6903	0.2936
2008	0.8811	0	0.9114	0.7328	0	0.1024	0.6263	0.1327
2009	0.7858	0	0.8723	0.4856	0	0.0283	0.4896	0.0485
2010	0.7433	0	0.8638	0.3604	0	0.0246	0.4435	0.0382
2011	0.6917	0	0.8508	0.2829	0	0.0295	0.4102	0.0398
2012	0.5769	0.0001	0.8104	0.2278	0	0.0448	0.3657	0.052
2013	0.5302	0.0013	0.8008	0.1701	0.0004	0.0798	0.3525	0.077
Average P	0.7784	0.2121	**0.8799**	**0.5640**	0.2120	0.2742	**0.5873**	0.2877

Diagnosed

Year	Base Model	Exhaustion of Susceptibles	ART Usage	Lack of Access to Care	ES & ART	ES & LAC	LAC & ART	ES & LAC & ART
2005	0.9998	0.9998	0.9998	0.9998	0.9998	0.9998	0.9998	0.9998
2006	0.8534	0.8534	0.8534	0.8534	0.8534	0.8534	0.8534	0.8534
2007	0.9847	0.8281	0.984	0.9847	0.984	0.9847	0.9999	0.984
2008	0.9215	0.0085	0.9176	0.9349	0.9176	0.8995	0.9828	0.9176
2009	0.8931	0	0.8862	0.9264	0.8862	0.8324	0.9753	0.8862
2010	0.8618	0	0.8507	0.9211	0.8507	0.7432	0.9665	0.8507
2011	0.8286	0	0.8109	0.916	0.8109	0.6429	0.9527	0.8109
2012	0.8064	0	0.7846	0.9217	0.7846	0.546	0.9461	0.7846
2013	0.762	0	0.7361	0.9134	0.7361	0.4328	0.9256	0.7361
Average P	0.8790	0.2989	**0.8693**	**0.9302**	**0.8693**	0.7705	**0.9558**	**0.8693**

Table 1. The likelihood of the data being observed in 100 stochastic simulations compared between the 7 scenarios. The 3 highest average probability over the 9 years is bolded.
