# Supplementary material for: Estimating epidemiological parameters of a stochastic differential model of HIV dynamics using hierarchical Bayesian statistics
Source: PLoS One. 2018 Jul 25;13(7):e0200126. doi: 10.1371/journal.pone.0200126 (PMC6059410; doi:10.1371/journal.pone.0200126)
Supplement: S1 Fig — Stochastic simulations for the combined cases. (A) Exhaustion of Susceptibles and lack of access to care. (B) Exhaustion of Susceptibles and ART. (C) Lack of access to care and ART. (D) Exhaustion of Susceptibles, lack of access to care, and ART. (PDF) [file pone.0200126.s006.pdf]

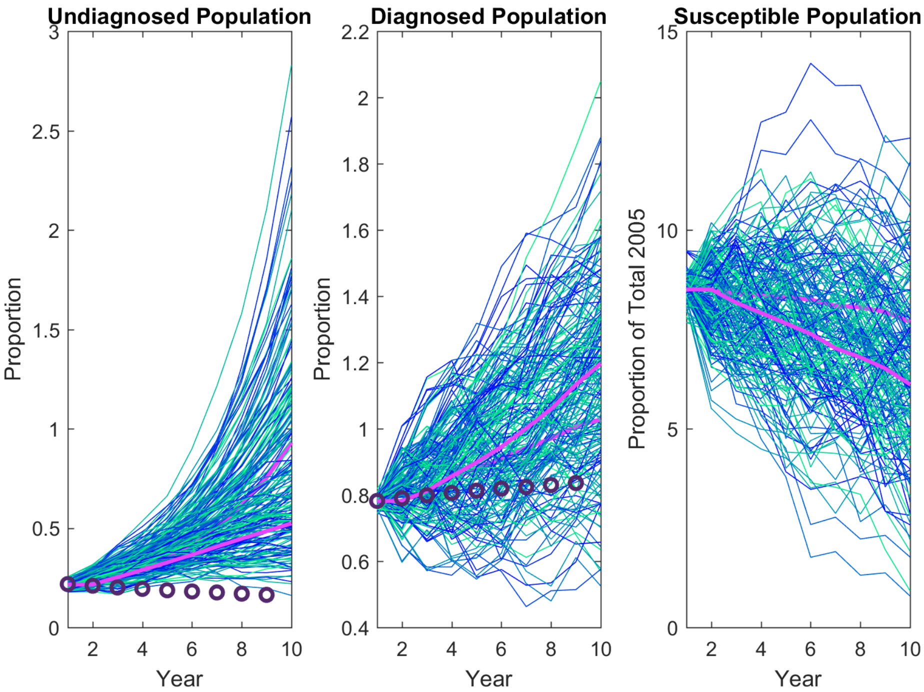

Fig 1. (A) Exhaustion of Susceptibles and ART.

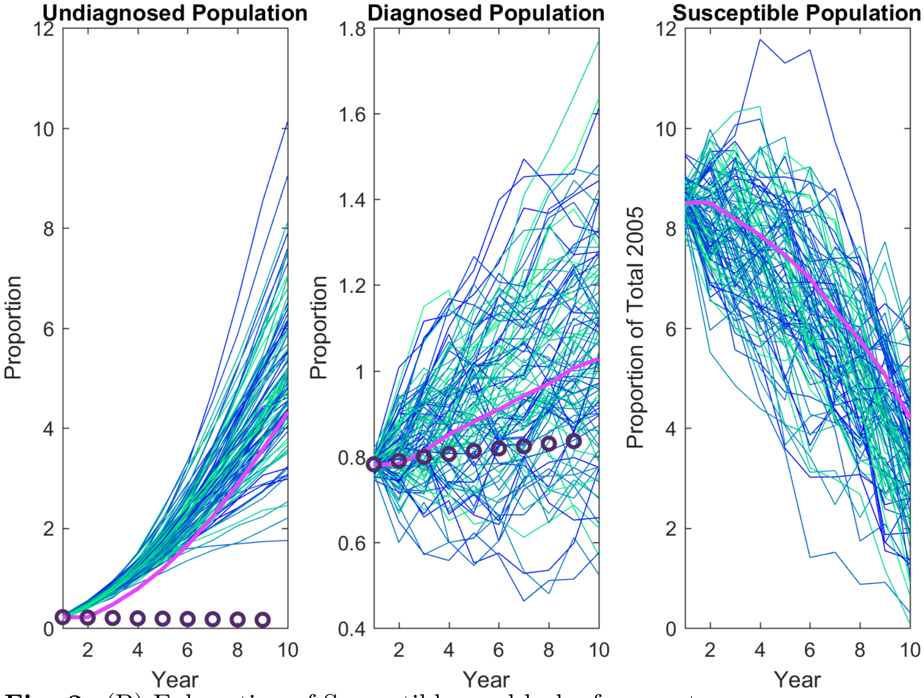

Fig 2. (B) Exhaustion of Susceptibles and lack of access to care.

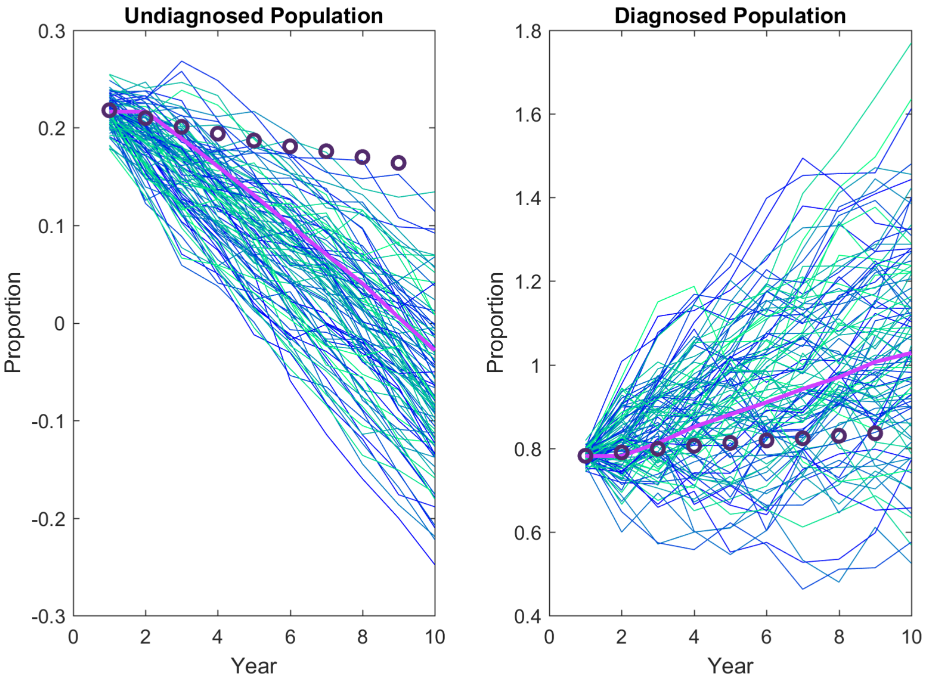

Fig 3. (C) Lack of access to care and ART.

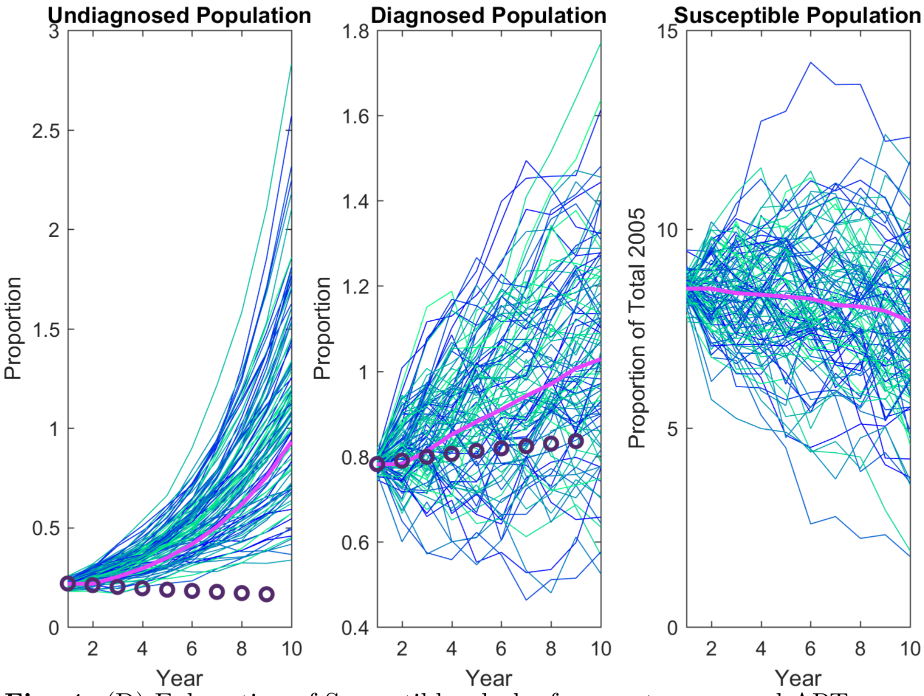

Fig 4. (D) Exhaustion of Susceptibles, lack of access to care, and ART.
